# Guy's Hospital

**Published:** 1902-01-18

**Authors:** 


### GUY'S HOSPITAL.

### THE RENOVATION AND BUILDING FUND, SPECIAL APPEAL.

The great hall at the Mansion House was filled with an influential gathering on Wednesday in support of the Appeal now being made for £180,000 towards a Renovation and Building Fund, and the raising of the ordinary income of the hospital to £60,000 per annum.

The Lord Mayor (Sir Joseph Dimsdale) presided, and in opening the proceedings said he had on many occasions been privileged to plead the cause of charity, but he had never risen with greater pleasure to advocate any cause than he did that day on behalf of Guy's Hospital. For that was an institution which was beneficial to the whole community. The rich were as much benefited by the labours of the students and demonstrators in the medical schools, and those of the doctors and nurses in the wards, as were the poor; the results of those labours advantaged not the hospital alone, nor even the Metropolis alone, but the whole Empire and the whole world shared in the results obtained. The expansion of the work of the hospital since its foundation nearly 200 years ago had been enormous. In 1724, in the time of Thomas Guy, 50 nurses were considered ample to carry out the requirements, while to-day 250 were held to be barely sufficient for the work; and indeed the work could not be done with that number unless the nurses and staff worked together as one for the alleviation of the suffering of mankind. And it was a regrettable fact that even with this expansion, there were beds closed which could not be opened unless the public provided the wherewithal. The hospital was absolutely essential to the welfare of the metropolis, and he could not believe that those who considered themselves merely trustees for the wealth with which they had been blessed would allow its beneficent activities to be curtailed. He would remind them that that hospital was almost unique among such institutions. From 1724 to 1880 it was absolutely self-supporting; but unfortunately its income was derived very largely from real property, and the depression in agriculture reduced that income by 50 per cent. That was the reason for one of the appeals they were making that day to restore the income of the hospital to its former level. The other appeal was to the people of London and of the country at large to come to their assistance and help to improve the condition of the building and make it worthy of one of the great hospitals of London. He need hardly refer to the fact that an institution which had existed the length and time that Guy's had must need a great deal of work done to the fabric and structure to keep it abreast of modern requirements. Unless it were up to date, with its schools in such a state of efficiency that students were eager to come in, and its wards in a thoroughly healthy and sanitary condition, it would better not exist at all. So they were pleading not only for £60,000 a year more income to keep it going, but they wanted a sum of £180,000 for capital outlay. Money must be spent not only on the renovation of the fabric and the wards, but on the operating theatres, the Medical College, the Nurses' Home, and in providing a first-class laundry, and in the sanitary arrangements generally. That sum would, of course, not be required all at once, so that if it were more convenient to spread donations over three or five years they would be equally useful and equally appreciated by the authorities of the hospital. The work the hospital was doing spoke for itself. They had 652 beds open for in-patients, and in 1900, 7,320 in-patients were received, while in 1901, the figures for which were just to hand, 7,811 in-patients were treated. The out-patients numbered 120,849 poor sufferers, while the maternity cases treated in the patients' own homes numbered 4,313. Those figures required no comment; and in addition they sent doctors and nurses trained in that magnificent institution throughout the kingdom and the Empire. Presiding here as London's chief magistrate, in the old Mansion House, that centre of the country's benevolence, he pleaded for that great hospital founded nearly 200 years ago by a fine old citizen of London; and he left it to those who were able to assist the poor who could not help themselves, to look for a far higher reward than any that could be given them this side of the grave.

The Treasurer (Mr. H. Cosmo Bonsor) then announced that letters of regret at inability to be present had been received from the Duke of Cambridge (senior governor of the hospital), Lord Salisbury, Lord Rosebery, and many others; and read the following letter from the Right Hon. A. J. Balfour, M.P.:—

10 Downing Street, S.W., January 10th, 1902.

My dear Bonsor,—I very deeply regret my enforced absence from the Mansion House meeting on Wednesday next. The immediate assembling of Parliament, however, renders that date peculiarly inconvenient, and the pressure of other work deprives me of any hope of being present. I particularly desired to take part in your proceedings, for I strongly feel that the occasion is a unique one, and that on the success of your efforts to collect funds depends in no small measure the future value of the work which it will be in the power of Guy's Hospital to perform in the service of humanity and of science.

The necessity of a capital expenditure of £180,000 is, I well know, imperative. Without a modern equipment this great institution cannot effectively carry on its beneficent work; and when it is remembered that this work has now for nearly two hundred years not only alleviated the sufferings of the poor, but contributed its full share to those marvellous advances in the science of healing and nursing by which the whole community, irrespective of class, so largely benefit, I cannot think that any difficulty should be found in providing the money.

With the earnest hope that London will not suffer the discredit of withholding this needful aid from one of its most admirable institutions.

I beg to remain, yours very sincerely,

(Signed) Arthur James Balfour.

Mr. Cosmo Bonsor, then addressed the meeting. He said the Lord Mayor had been so good as to lend the Mansion House for the purpose of an appeal in favour of the funds of Guy's Hospital. In thanking him for the speech which he had just delivered, and for the use of that hall, he had on behalf of the governors to state that a period in the conduct of their trusteeship had arrived when they required the cordial support and the financial assistance of the citizens of London. The Lord Mayor had well reminded the meeting that Guy's Hospital was founded by a citizen of London; that it was founded for the purpose of relieving distressed humanity, and he believed that the principal object which influenced Thomas Guy in his benevolence was that no sick person whose malady might seem incurable should be turned out of the hospital for the purpose of dying. That tradition had been one of many connected with that hospital which appealed forcibly to all kind-hearted people. It was, he thought, in 1882 or 1883 that the question of the hospital's finances was seriously taken in hand by the governors. The question they had to consider was, whether they were merely trustees for the distribution of the endowments left them by Thomas Guy, which endowments by reason of fallen rentals were reduced to a half of their one time value, or whether they had a further duty to the public, in the locality in which they were placed, of providing to the best of their means and opportunity sufficient accommodation for the sick poor. He need hardly say that the answer to the question was never in doubt. The governors in the first place collected among themselves and their personal friends a sum of £12,000 to relieve the embarrassment of the time, and shortly afterwards they made a public appeal, principally to the bankers and merchants of London, for support. This appeal met with a warm response, and sufficient funds were thus raised to enable the governors to keep a reduced number of beds open until the year 1895. In the autumn of that year his predecessor, Mr. Lushington, made a further appeal to the public for the purpose of replacing the loss of income which had occurred through the decreased agricultural rental coming to the hospital. The late Mr. Gladstone, who was one of the Senior Governors, wrote an eloquent letter, to which they attributed gifts of £30,000, and, having started the appeal well on its way, Mr. Lushington resigned his position as treasurer at the same time that Lord Aldenham resigned his position as president of the hospital in favour of the Prince of Wales, now his Majesty the King. As president of the hospital the King signalised his acceptance of the office by personally leading a new movement having for its purpose the re-endowment of the hospital to the extent of its losses, and the response of the public was exceedingly generous, but insufficient, to finally rescue the hospital from its difficulties. His Majesty was afterwards gracious enough to open one of the new medical school buildings which had been built by the medical staff, out of their own resources, and he expressed a strong wish that the governors of the hospital should make an effort to re-open all the beds that were closed. On behalf of the governors, the speaker, as treasurer, undertook that obligation, and all their efforts since 1896 and the extra expenditure that had been undertaken had been directed to the one end of throwing open the whole of the 650 beds to the service of the sick poor. To open 150 closed beds under modern conditions of medical science and professional nursing necessarily required a heavy expenditure. In the early days of the hospital's history 50 nurses were considered sufficient for the needs of the patients, messengers being sent out into the neighbourhood to get voluntary assistance when necessary. To-day they had 230 sisters and nurses, and every nurse in charge of a sick patient must be thoroughly trained and thoroughly efficient. Then the older wards of the hospital had to be remodelled, for the sanitary and general arrangements which sufficed 180 years ago, could not be contemplated by any medical staff as wholesome at the present day. To this renovation of wards was largely due the acceleration in the cure or relief of surgical in-patients which ten years ago took on an average 25 days and was now reduced to 19 days. Again, the washing of the linen necessary to an establishment of that description under modern requirements was very different from what it was even within living memory. Moreover, the old laundry was worn out. The expenditure on a nurses' home capable of lodging the nurses required for the care of 650 in-patients was of necessity a large one, and the Governors would have hesitated before embarking on such an enterprise had it not been for the generosity of the late Mr. Henry Raphael, who called one cold morning in January, 1898, and after having visited the attics in which the nurses were lodged, and inspected an adjoining site where it was possible to build a nurses' home, gave a cheque for £20,000 to that object. That donation was the first direct encouragement which the governors received towards the outlay requisite to bring Guy's into an absolutely efficient condition. Since then the governors had received many encouragements. The medical and surgical staff of the hospital, acting in cordial co-operation with the governors, had been good enough to subscribe out of their own pockets the large sum of £1,800. Many of the governors of the hospital had not only given large subscriptions, but at times, when he, as treasurer, with debt staring them in the face, had felt it his duty to say that certain of the works should be stopped, some of them had come forward as individuals to undertake particular improvements. And the poor recipients of the charity had done what they could. In-patients and out-patients, either directly or indirectly, had contributed £500 during the last twelve months, absolutely voluntarily, on leaving the hospital after receiving its benefits. The press has been most generous, and besides these encouragements he has received an intimation that many men who were working on the railways in close proximity to the hospital were making a private subscription among themselves for the purpose of assisting, and through the great kindness of Mr Beerbohm Tree, a special evening performance would be given at Her Majesty's Theatre in the spring, in what they all hope would be a most crowded season. A good friend of the hospital, the Countess of Bective, was most generously placing her great influence and organising abilities at their disposal in connection with that performance, and they looked forward to the occasion being worthy of the cause and of the Coronation year. Those were the conditions under which they, as governors of the hospital, came before the citizens of London. Their requirements were, firstly, a sum of £180,000 to meet the cost of those works to which reference had been made, and, secondly, for an additional income of not less than £14,000 per annum, and H.R.H. the Prince of Wales has been the first to subscribe to the latter necessity. They made a direct appeal to the bankers and merchants, the traders, and also to the City Guilds, to assist a great institution which for so many generations had carried on its work of charity. They asked them to do the same as the press, who had sent representatives to the hospital, to come and see for themselves the work that was being carried on. He believed he had said enough for the citizens of London to recognise that it ought to be impossible to allow a hospital such as Guy's, standing within a stone's throw of the greatest, richest, and best governed city in the world, a hospital, too, with such noble traditions, to suffer neglect or to continue in a state of financial embarrassment.

Mr. LEOPOLD DE ROTHSCHILD moved, and Hon. ALLAN GIBBS, M.P. seconded the following resolution :—

"That this meeting having heard the speech of the Right Hon. the Lord Mayor and the statement of the treasurer showing the present urgent financial needs of Guy's Hospital, earnestly commends the same to the support of the citizens of London and to the benevolent public throughout the country, in the hope that sufficient assistance may be immediately rendered to enable the Governors to maintain the work of that great institution in a state of completeness and efficiency."

This was supported by Mr. A. C. Cole, Mr. Causton, M.P. for Southwark, and carried.

Mr. BONSOR then read a list of donations received to date, amounting to about £64,000.

Dr. FREDERICK TAYLOR, the senior physician, moved a vote of thanks to the Lord Mayor, which was seconded by Mr. HOWSE, the senior surgeon (President of the Royal College of Surgeons), and adopted; after which the proceedings terminated.

